# The Mediating Role of Emotion Regulation Strategies in the Relationship Between Big Five Personality Traits and Anxiety and Depression Among Chinese Firefighters

**DOI:** 10.3389/fpubh.2022.901686

**Published:** 2022-06-03

**Authors:** Yanqiang Tao, Xiangping Liu, Wenxin Hou, Haiqun Niu, Shujian Wang, Zijuan Ma, Dan Bi, Liang Zhang

**Affiliations:** ^1^Faculty of Psychology, Beijing Key Laboratory of Applied Experimental Psychology, Beijing Normal University, Beijing, China; ^2^School of Psychology, Nanjing Normal University, Nanjing, China; ^3^School of Psychology, South China Normal University, Guangzhou, China; ^4^Yichun 1st High School, Yichun, China; ^5^College of Education for the Future, Beijing Normal University at Zhuhai, Zhuhai, China; ^6^Student Mental Health Education Center Northeast Agricultural University, Harbin, China

**Keywords:** firefighters, emotion-regulation strategies, personality traits, anxiety, depression

## Abstract

Identification of protective factors to prevent firefighters' anxiety and depression is meaningful. We explored whether emotion-regulation strategies mediate the relationship between personality traits and anxiety and depression among Chinese firefighters. Approximately, 716 Chinese firefighters were recruited and completed the Emotion Regulation Questionnaire (ERQ), Self-Rating Anxiety Scale (SAS), Self-Rating Depression Scale (SDS), and Big Five Inventory−2 (BFI-2) Scale. Results (*N* = 622) indicated that only negative emotionality traits could predict anxiety symptoms. Meanwhile, the multilevel mediation effect analyses showed that conscientiousness through cognitive reappraisal could reduce anxiety and depression symptoms in Chinese firefighters. Our findings clarify Chinese firefighters' underlying emotion-regulation process between personality traits and anxiety and depression. Implications, limitations, and future directions are discussed.

## Introduction

Numerous studies have shown that firefighters are frequently exposed to traumatic events and stressful situations, increasing their vulnerability to developing psychiatric problems, such as anxiety (1, 2) and depression (3, 4). Anxiety symptoms refer to excessive nervousness-related emotional and behavioral responses (e.g., avoidance) and related cognitive patterns (5). Depression is characterized by widespread negative emotions, greatly impacting cognitive, emotional, social, and occupational functions (5). A study on the mental health of the firefighters in China revealed that the overall mental health of the firefighters was at an intermediate level, and 5% of them were terrible (6). Similarly, another study also found that at least 4% of Chinese firefighters had psychological problems, including anxiety, depression, and low self-esteem, especially young firefighters (7). As emergent workers, effective interventions to avoid firefighters' depression and anxiety symptoms are meaningful. Notably, early interventions may help shun potential morbidity (8, 9). Hence, it is essential to identify protective factors to prevent anxiety and depression for firefighters and improve their wellbeing.

### Personality Traits and Anxiety and Depression Symptoms

The Five-Factor Model (FFM) of personality seems to be the most prominent and influential in contemporary psychology (10–12), which could define individual differences in thoughts, feelings, and actions patterns at different dimensions (13). The FFM assesses five traits: neuroticism (i.e., negative emotionality), extraversion (i.e., positive emotionality), openness to experience (open-mindedness), agreeableness, and conscientiousness (14, 15).

Previous studies have revealed that negative emotionality is a general contributor to anxiety disorders (16–18), and persons with depressive disorders tend to be highly neuroticism (19). Besides, extraversion is uniquely related to social anxiety (17), with agreeableness (20) showing consistent negative associations with anxiety (21, 22) and depression symptoms (16). Pointedly, less is known about the role of conscientiousness and openness in anxiety and depression symptoms. Although several studies have focused on the relationships between personality traits and anxiety and depression (23), not all personality traits are associated with anxiety and depression (18, 19). Moreover, information about this linkage in Chinese firefighters has been sparsely documented and, whether there are personality traits associated with anxiety and depression, is still unknown from previous studies. We hypothesized that variations in personality traits would affect their relationship with firefighters' anxiety and depression symptoms (H1).

### Mediating Role of Emotional Regulation

Emotion regulation is defined as the process through which people can adjust or express their emotions and experience using cognitive or behavioral strategies (24, 25). As an effective coping strategy, emotional regulation has been widely used in improving individual mental health (26). There are many emotion regulation strategies (27), such as expressive suppression, cognitive reappraisal, acceptance (28), distraction (29), and rumination (30). The most commonly researched emotion regulation strategies are cognitive reappraisal and expressive suppression. Cognitive reappraisal reflects tendencies to think about the situation differently to modify emotional impact (31). Expressive suppression refers to the attempts to suppress thoughts and emotions associated with the situation and emotional expression (32).

Cognitive reappraisal yields effective benefits by increasing positive emotions and crumbling negative ones (33). Higher cognitive reappraisal frequencies are specifically associated with higher life satisfaction (34, 35), self-esteem (36), and wellbeing (37). Expressive suppression belongs to the process of model's response modulation category, which happens after the emotion has been generated (38). Compared to cognitive reappraisal, expressive suppression aims to deal with external or behavioral emotional responses with little effect on controlling internal emotional responses. From previous research, the use of expressive suppression to manage negative emotions, such as sadness or anxiety, has been proved to intensify negative emotions, while the use of expressive suppression to manage positive emotions, such as happiness, has been shown to inhibit positive emotions experience (39, 40).

Individual differences reflected in variations of personality dimensions contribute to emotional reactivity and regulation (41). For instance, individuals with high scores of extraversion, agreeableness, openness to experience, and conscientiousness use more communication to enhance positive emotions and regulate negative emotions. While people with high neuroticism are more likely to depend on other persons for social modeling and mollification (42). Besides, it has been found that low extraversion is a possible precursor of using emotional suppression to adjust emotions, while neuroticism has a moderate negative correlation with cognitive reappraisal (43). Overall, people with different personality traits would take different emotional regulation strategies. Thus, we hypothesized that the relationship between five personality traits and cognitive reappraisal and expressive suppression differed from each other (H2).

According to Barlow (44), mental disorders are emotional ailments. Hence, numerous research have also established a link between emotion regulation and psychiatric symptoms (45, 46). Specifically, a link has been established between emotional dysregulation and depressive symptoms (47). Another study discovered that emotional regulation deficiencies were associated with suicidal ideation (48). On the other side, however, studies show that using emotional suppression may entice internal attack, and, thus, people applying emotional suppression are more prone to experience negative effects or even depressive symptoms (43). In other words, different emotion regulation strategies may prevent or even elicit mental problems. Because firefighters are trained people with competencies and abilities in managing dangerous situations, they may have stronger control over their emotions, especially negative ones (49). Thus, we hypothesized that both emotional regulation strategies could negatively predict firefighters' anxiety and depression symptoms (H3).

Moreover, in the light of the biopsychosocial model in health psychology (50), coping strategies can mediate the relationship between dispositional characteristics, such as personality traits and health-related outcomes. Thus, it is vital to elucidate the factors that might mediate the association between personality traits and anxiety and depression symptoms. Taken all together, we hypothesized that cognitive reappraisal and expressive suppression could play a mediating role in the relationship of personality traits – anxiety and depression symptoms (H4).

In summary, the present study first examined the relationship between different personality traits and anxiety and depression symptoms in Chinese firefighters. Second, we examined the extent to which emotion-regulation strategies might mediate the influence of different personality traits on anxiety and depression symptoms and expected a negative correlation between personality traits and anxiety and depression symptoms except for negative emotionality.

## Materials and Methods

### Participants

The current study recruited 716 full-time male professional firefighters in China voluntarily. After eliminating multivariate variables' outliers, the final participant sample was 622 (*Mean*_age_ = 26.25, *SD*_age_ = 3.12). The mean working experience was 29.36 months. The ethical committees of all the authors' universities approved this study. The informed consent was obtained from the participants before they took part in either assessment.

### Measures

#### Emotion Regulation Questionnaire

The ERQ (51) is a 10-item self-report and ranges from 1 to 7 (1 = *strongly disagree*; 7 = *strongly agree*), which measures an individual's habitual use of expressive suppression and cognitive reappraisal to regulate emotion. The expressive suppression subscale comprises 4 items, and the cognitive reappraisal subscale includes 6 items. Subscales were calculated based on mean values, with higher scores indicating higher frequencies of usage. The Chinese version of ERQ was validated for both adults and adolescents, and it demonstrated a good Cronbach's alpha value (0.73 for reappraisal and 0.71 for suppression) (52). In the current study, Cronbach's alpha of cognitive reappraisal and suppression subscales were 0.85 and 0.70, respectively. The questionnaire had good validity [χ^2^/*df* = 3.98, CFI = 0.97, NFI = 0.96, TLI = 0.95, RMSEA (90% *CI*) = 0.07 (0.06–0.08)] in the current study.

#### Big Five Inventory−2

The BFI-2 is a 60-item self-report measure of personality traits (53) and comprises five subscales: extraversion, agreeableness, conscientiousness, negative emotionality, and open-mindedness, and each subscale has 12 items, respectively. Each item is rated on a 5-point Likert scale (1 = *strongly disagree*; 5 = *strongly agree*). Subscales were calculated by mean value, with higher scores indicating more inclined toward the specific personality.

The present study used a Chinese version of the BFI-2 questionnaire to evaluate personality traits (15). The current study showed good internal consistency, Cronbach's alpha value = 0.74,0.84,0.85,0.82, and 0.76 for extraversion, agreeableness, conscientiousness, negative emotionality, and open-mindedness, respectively. The questionnaire had good validity [χ^2^/*df* = 4.11, CFI = 0.71, NFI = 0.65, TLI = 0.69, RMSEA (90% *CI*) = 0.07 (0.06 −0.07)] in the current study.

#### Self-Reported Anxiety and Depression Symptoms

Anxiety and depression symptoms were assessed using the Self-Rating Anxiety Scale (SAS) and the Self-Rating Depression Scale (SDS), respectively, which were designed by Zung (54, 55) to quantify the degree of anxiety and depression symptoms. Although SAS and SDS cannot be used to diagnose anxiety and depression clinically, they are good tools for evaluating and screening anxiety and depression symptoms and determining the cohort of high-risk patients with anxiety and depression. SAS and SDS scales are both 20-item self-report assessment instruments. All subscales were calculated by mean value, with higher scores indicating more inclination toward anxiety or depression.

The Chinese versions have been validated in epidemiological surveys (56, 57). The current study showed good internal consistency, Cronbach's alpha = 0.93, and 0.92 for anxiety and depression, respectively. The questionnaire had good validity for anxiety [χ^2^/*df* = 3.90, CFI = 0.94, NFI = 0.92, TLI = 0.93, RMSEA (90% *CI*) = 0.07 (0.06 −0.07)] and depression [χ^2^/*df* = 4.89, CFI = 0.92, NFI = 0.90, TLI = 0.90, RMSEA (90% *CI*) = 0.08 (0.07–0.09)], respectively.

### Data Analysis

In the present study, all analyses were calculated in *R*4.1.1 (58). Initially, we performed data cleaning and deleted outliers using the *boxplot.stats* function (58). Then, a common method deviation test, description analyses, and normal distribution were done using the *psych* package (59). The correlation matrix table was made by *apaTables* (60). Finally, we conducted the multiple level mediation analysis by *lavaan* (61).

## Results

### Common Method Deviation Test

The Harman single factor test showed that the eigenvalues of eighteen-eight factors were more outstanding than one without rotation, and the explanatory variation of the first factor was 21.784%, which was lower than the critical value of 40% (62). Therefore, there was no obvious common methodological bias in this study.

### Descriptive Statistics and Correlations Among Main Measures

The means, standard deviations (*SD*), and spearman correlations between main variables are shown in Table 1. Results indicated that cognitive reappraisal and expressive suppression were significantly and negatively correlated with anxiety and depression (*p* < 0.01). Conversely, cognitive reappraisal and expressive suppression were significantly and positively correlated with negative emotionality (*p* < 0.01). Similarly, both anxiety and depression were significantly and negatively correlated with extraversion, agreeableness, conscientiousness, and open-mindedness (*p* < 0.01). Furthermore, both anxiety and depression were significantly and positively correlated with negative emotionality (*p* < 0.01).

**Table 1:** Means, standard deviations, and pearson correlations.

	***M***	***SD***	**1**	**2**	**3**	**4**	**5**	**6**	**7**	**8**	**9**
1. Cognitive reappraisal	4.03	0.95	1								
2. Expressive Suppression	4.72	1.08	0.71**	1							
3. Depression	2.28	0.32	−0.21**	−0.15**	1						
4. Anxiety	1.87	0.34	−0.35**	−0.27**	0.41**	1					
5. Extraversion	3.39	0.55	0.32**	0.23**	−0.12**	−0.40**	1				
6. Agreeableness	4.07	0.62	0.32**	0.29**	−0.11**	−0.41**	0.59**	1			
7. Conscientiousness	3.97	0.66	0.37**	0.32**	−0.16**	−0.45**	0.67**	0.79**	1		
8. Negative Emotionality	2.33	0.66	−0.31**	−0.25**	0.13**	0.49**	−0.68**	−0.73**	−0.76**	1	
9. Open-Mindedness	3.55	0.59	0.33**	0.26**	−0.18**	−0.36**	0.66**	0.61**	0.68**	−0.60**	1

M and SD are used to represent mean and standard deviation, respectively. ^**^ indicates p <0.01.

### Multiple Mediation Analysis

**Figure 1:**
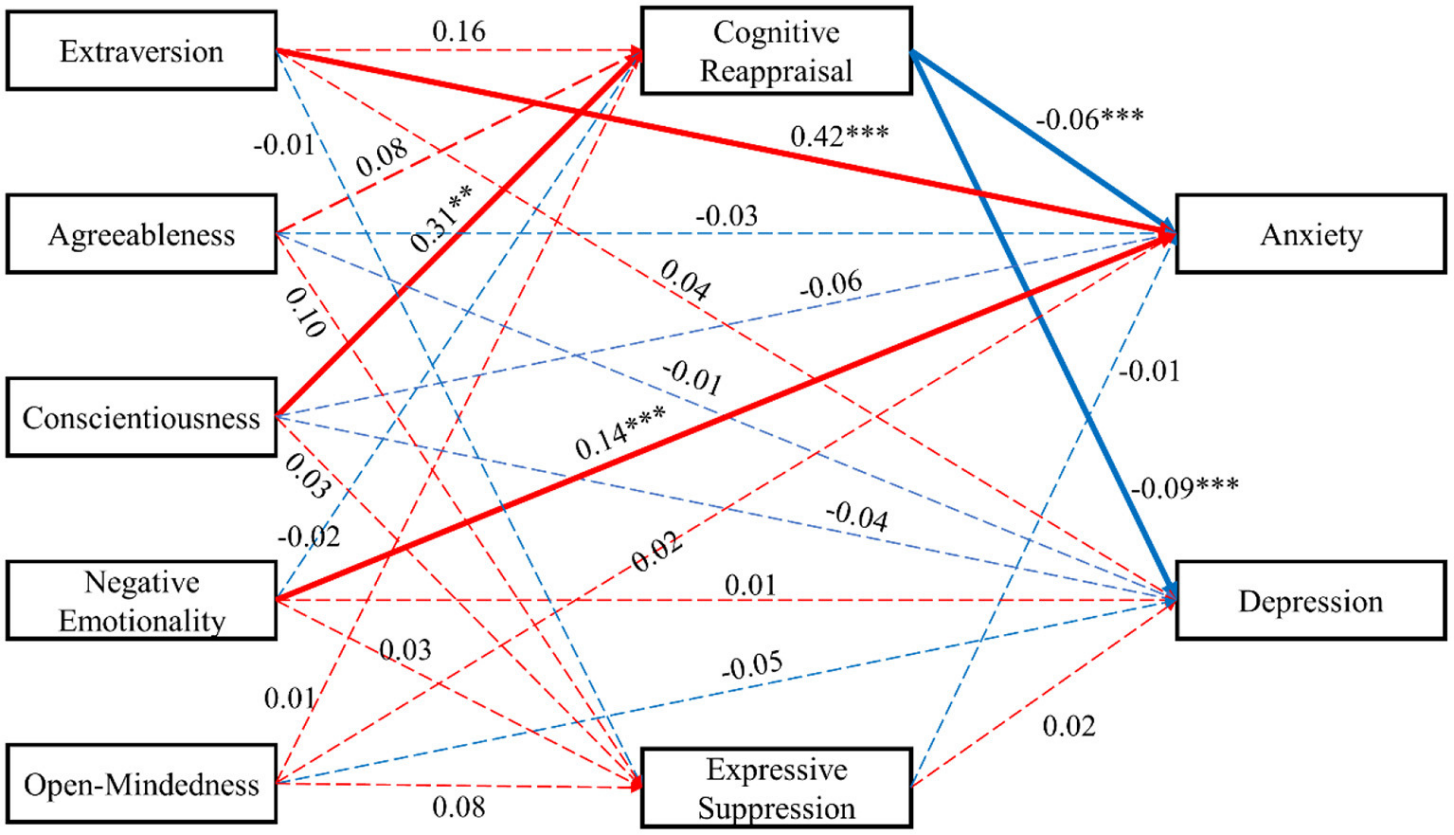
The model of mediating role of emotion regulation strategies between personality traits and anxiety and depression symptoms. The red line indicates the positive regression coefficient. The blue line indicates the positive regression coefficient. The solid line indicates that the regression coefficient is significant (*p* < 0.05). The dashed line indicates that the regression coefficient is insignificant (*p* > 0.05). ** indicates *p* < 0.01. *** indicates *p* < 0.001.

We used the package of *lavaan* to test multiple level mediations (see Figure 1). The result indicated that the model fitted the data well [χ^2^/*df* = 3.364, *CFI* =0.997, *NFI* = 0.996, *TLI* = 0.929, *RMSEA* (90% *CI*) = 0.062 (0.000–0.139)]. Path analysis revealed that conscientiousness significantly and positively predicted cognitive reappraisal (β = 0.31, *p* < 0.01) and expressive suppression (β = 0.42, *p* < 0.001). By contrast, other personality traits could not significantly predict cognitive reappraisal and expressive suppression (*p* > 0.05). Furthermore, path analysis revealed that only negative emotionality could significantly predict anxiety symptoms (β = 0.14, *p* < 0.001). As the mediator variable, only cognitive reappraisal could significantly and negatively predict anxiety (β = −0.06, *p* < 0.001) and depression symptoms (β = −0.09, *p* < 0.001), conversely; expressive suppression was not (*p* > 0.05).

We conducted bias-corrected bootstrap tests (Created 5,000 bootstrap samples, 95% confidence interval) to evaluate the significance of the direct effects shown in Table 2. As the bias-corrected bootstrap tests mentioned, if the 95% confidence interval of the direct path coefficient does not include 0, it is suggested that the direct path is significant. Table 2 shows the bias-corrected bootstrap test results. Results confirmed that indirect pathways from conscientiousness to anxiety and depression symptoms at cognitive reappraisal strategies were significant. In sum, cognitive reappraisal strategies fully mediated the relation between conscientiousness, anxiety, and depression among Chinese firefighters.

**Table 2:** Bias-corrected bootstrap test of mediating effects.

**Paths**	**Standardized β (*SE*)**	**Standardized** **95%** ***CI***	
		**Low**	**High**
**Direct paths**			
Extraversion–Anxiety	−0.040 (0.031)	−0.102	0.021
Agreeableness–Anxiety	−0.032 (0.034)	−0.100	0.033
Conscientiousness–Anxiety	−0.059 (0.035)	−0.128	0.009
Negative Emotionality–Anxiety	0.141*** (0.031)	0.076	0.201
Open-Mindedness–Anxiety	0.024 (0.031)	−0.034	0.086
Extraversion–Depression	0.040 (0.035)	−0.027	0.110
Agreeableness–Depression	−0.006 (0.032)	−0.071	0.055
Conscientiousness–Depression	−0.040 (0.037)	−0.110	0.034
Negative Emotionality–Depression	0.009 (0.030)	−0.051	0.066
Open-Mindedness–Depression	−0.048 (0.035)	−0.116	0.021
**Indirect paths**			
Extraversion–Cognitive Reappraisal–Anxiety	−0.010 (0.007)	−0.028	0.002
Agreeableness–Cognitive Reappraisal–Anxiety	−0.005 (0.006)	−0.019	0.007
Conscientiousness–Cognitive Reappraisal–Anxiety	−0.019* (0.008)	−0.039	−0.006
Negative Emotionality–Cognitive Reappraisal–Anxiety	0.001 (0.006)	−0.010	0.013
Open-Mindedness–Cognitive Reappraisal–Anxiety	−0.006 (0.007)	−0.021	0.005
Extraversion–Cognitive Reappraisal–Depression	−0.014 (0.011)	−0.039	0.003
Agreeableness–Cognitive Reappraisal–Depression	−0.007 (0.010)	−0.028	0.010
Conscientiousness–Cognitive Reappraisal–Depression	−0.027* (0.012)	−0.056	−0.009
Negative Emotionality–Cognitive Reappraisal–Depression	0.002 (0.008)	−0.014	0.019
Open-Mindedness–Cognitive Reappraisal–Depression	−0.009 (0.009)	−0.031	0.007
Extraversion–Expressive Suppression–Anxiety	0.000 (0.002)	−0.004	0.005
Agreeableness–Expressive Suppression–Anxiety	−0.001 (0.003)	−0.011	0.002
Conscientiousness–Expressive Suppression–Anxiety	−0.003 (0.006)	−0.017	0.007
Negative Emotionality–Expressive Suppression–Anxiety	0.000 (0.002)	−0.006	0.002
Open-Mindedness–Expressive Suppression–Anxiety	−0.001 (0.002)	−0.008	0.002
Extraversion–Expressive Suppression–Depression	0.000 (0.003)	−0.008	0.006
Agreeableness–Expressive Suppression–Depression	0.002 (0.004)	−0.002	0.016
Conscientiousness–Expressive Suppression–Depression	0.007 (0.009)	−0.008	0.028
Negative Emotionality–Expressive Suppression–Depression	0.001 (0.003)	−0.003	0.009
Open-Mindedness–Expressive Suppression–Depression	0.001 (0.004)	−0.002	0.015

^*^Indicates p <0.05.

^***^Indicates p <0.001.

## Discussion

To sum up, the present study applied a large sample of firefighters to reveal the machine of emotion regulation strategies between personality traits and anxiety and depression symptoms. Several findings are worth discussing.

As an essential psychological state, negative emotionality is a general contributor to anxiety disorders (16–18), which could positively predict anxiety (17), and the present study also demonstrated it (A part of Hypothesis 1 was supported). As we know, firefighters are always in a readiness state, and the long term of dealing with uncertainties may make firefighters worried and afraid of the terrible events. It is precise because of the adverse effects of constant uncertainties and confrontation that firefighters with neurotic traits are prone to anxiety. Hence, correctly evaluating and handling negative emotions is essential for firefighters. In contrast to previous research (63), a high level of negative emotionality could not predict depressive symptoms in the present study, even if patients who undergo negative emotional states could significantly predict depressive symptoms (64). The particularity of firefighters' occupations shows them a more stable and rational mental state and better self-psychological adjustments when dealing with negative events.

Another general trait, extraversion or positive emotionality, has shown negative associations with a generalized anxiety disorder (65), which was not confirmed in the present study (A part of Hypothesis 1 was not supported). When confronted with unfavorable events or emotional arousal, extroverted personality groups are more likely to engage in conservative decision-making, which cannot improve decision-making (66). While those with an extroverted personality are more likely to be satisfied and wellbeing (67), it should not be overlooked that, for the firefighters, the inability to deal with negative events calmly and comfortably frequently results in psychological problems, such as anxiety. Moreover, the present study found that positive emotionality could not be a valid predictor factor for depression, which is relevant to firefighters' occupations. On the other hand, with the organization's support (68) and frequent internal communication (69), the study found that the depression scores of the firefighters surveyed were low.

Conscientious firefighters were more likely to be disciplined, organized, and persistent about events in their daily lives. Indeed, in this study, we found that firefighters' conscientiousness was a significantly positive predictor for cognitive reappraisal (A part of Hypothesis 2 was supported), suggesting that firefighters with high level of conscientiousness tend to initiate reflection, summary and render emotional adjustment methods automatically (70). The analysis of mediating effects showed that firefighters' conscientiousness could influence anxiety and depression through cognitive reappraisal strategies instead of expressive suppression strategies (A part of Hypothesis 4 was supported). Previous research indicated that newly recruited firefighters showed significantly less depression than experienced firefighters (71), indicating that the experience of participating in fire rescues was also a vital predictor. We recruited firefighters with an average of 29.36 months of experience in fire rescue work for this study, implying that prolonged experience alters firefighters' emotional expression strategies.

It is beneficial for individuals to use more cognitive reappraisal strategies to change their attitudes and opinions about negative events (72), especially those who easily experience negative emotions. Cognitive reappraisal plays a positive role in predicting anxiety and depression in our current study, which is consistent with the previous study (73). Firefighters face many acute negative events and failures that can induce anxiety, depression (74), PTSD (75), and even suicide (76). In addition, firefighters with lower anxiety and depression are adept at using appropriate cognitive reappraisal strategies. As an essential risk factor, cognitive bias significantly influences firefighters' psychological resilience, which is substantially related to self-encouragement (72). It also provides us with an idea of correct and timely cognitive intervention that we should consider for firefighters in the future, especially for conscientious firefighters. Hence, good cognitive intervention is essential to predict the mental health status of firefighters (A part of Hypothesis 3 was supported).

However, expressive suppression strategies did not predict anxiety and depressive symptoms (A part of Assumption 3 is not verified). Specifically, expressive suppression strategies can moderate events that have occurred, while cognitive reappraisal can moderate events that have not occurred. A cross-cultural comparison study between European Americans and Hong Kong Chinese revealed that expressive suppression was associated with adverse psychological functioning for European Americans but not for Chinese participants (77), which implies that cultural context should be considered in understanding the emotional consequences of suppression strategies (78, 79). In China, the community has great respect for firefighters and communicates with firefighters frequently. Despite regular exposure to catastrophic and traumatic events, a high level of organizational and social support for the firefighting group has resulted in fewer dependencies on expression suppression strategies to regulate emotions and decreased depressive mood (80).

Several limitations should be considered in the current study. First, as a cross-sectional and correlational research design, our results are inadequate, prohibiting determining causality and whether the impact is stable over time. Future research should examine these constructs' temporal and reciprocal associations using a long-term cross-lag design, experimental methods, or network analysis. Secondly, more emotion regulation strategies (e.g., acceptance, distraction, rumination) should be included in future research work because individuals often adopt more emotion regulation strategies simultaneously in their daily lives.

## Conclusion

First, the current study extended the research on the relationship between different personality trait dimensions and anxiety and depression symptoms in firefighters. Additionally, the significant direct effect suggests that researchers must pay attention to firefighters with negative emotional traits. Second, cognitive reappraisal fully mediated the association between the conscientiousness trait and anxiety and depression symptoms. These results provide preliminary evidence supporting that conscientiousness is associated with low levels of anxiety and depression by using more cognitive reappraisal strategies. For firefighters, managers should guide them through utilizing effective cognitive reappraisal in their daily working lives.

## Data Availability Statement

The raw data supporting the conclusions of this article will be made available by the authors, without undue reservation.

## Ethics Statement

The studies involving human participants were reviewed and approved by Northeast Agricultural University. The patients/participants provided their written informed consent to participate in this study.

## Author Contributions

XL: study design. LZ and DB: data collection. YT: analysis, interpretation, and drafting of the manuscript. ZM, WH, HN, and SW: critical revision of the manuscript. All authors contributed to the article and approved the submitted version.

## Conflict of Interest

The authors declare that the research was conducted in the absence of any commercial or financial relationships that could be construed as a potential conflict of interest.

## Publisher's Note

All claims expressed in this article are solely those of the authors and do not necessarily represent those of their affiliated organizations, or those of the publisher, the editors and the reviewers. Any product that may be evaluated in this article, or claim that may be made by its manufacturer, is not guaranteed or endorsed by the publisher.
